# Emerging Roles of Long non-coding RNAs in The Tumor Microenvironment

**DOI:** 10.7150/ijbs.44420

**Published:** 2020-05-18

**Authors:** Lisha Zhou, Yingying Zhu, Dongsheng Sun, Qiang Zhang

**Affiliations:** 1 Taizhou University hospital, Taizhou University, Taizhou, Zhejiang, 318000, China; 2 Taizhou Municipal Hospital, Taizhou University, Taizhou, Zhejiang, 318000, China

**Keywords:** long non-coding RNA, tumor microenvironment, immune cells, cancer-stem cells, cancer-associated endothelial cells, cancer-associated fibroblasts

## Abstract

Long non-coding RNAs (lncRNAs) are a diverse class of longer than 200 nucleotides RNA transcripts that have limited protein coding capacity. LncRNAs display diverse cellular functions and widely participate in both physiological and pathophysiological processes. Aberrant expressions of lncRNAs are correlated with tumor progression, providing sound rationale for their targeting as attractive anti-tumor therapeutic strategies. Emerging evidences support that lncRNAs participate in tumor-stroma crosstalk and stimulate a distinctive and suitable tumor microenvironment (TME). The TME comprises several stromal cells such as cancer stem cells (CSCs), cancer-associated endothelial cells (CAEs), cancer-associated fibroblasts (CAFs) and infiltrated immune cells, all of which are involved in the complicated crosstalk with tumor cells to affect tumor progression. In this review, we summarize the essential properties and functional roles of known lncRNAs in related to the TME to validate lncRNAs as potential biomarkers and promising anti-cancer targets.

## Introduction

Long noncoding RNAs (lncRNAs) are a large class of transcribed RNA molecules that are greater than 200 nucleotides in length and have limited protein coding potential for the lack of functional open readings 1, 2. In term of functions of lncRNAs, guide, decoy, signaling and scaffold functions have been identified 3. Guide lncRNAs modulate gene expression via recruiting chromatin modifying enzymes to specific genomic regions, while decoy lncRNAs sequester transcription factors away from chromatin. Signaling lncRNAs serve as molecular signals and integrate with specific signaling pathways or events. Scaffold lncRNAs act as platforms to recruit multiple proteins to assemble functional units, such as ribonucleoprotein complexes, and regulate gene expression. To date, lncRNAs have been found to actively participate in the regulation of various aspects of tumor development, including growth, metastasis and recurrence 4-6. Indeed, lncRNAs can function as oncogenes or tumor suppressors, and aberrant expressions are linked to various human cancers, such as prostate cancer 7, colorectal cancer 8 and hepatocellular carcinoma 9. Notably, like circulating miRNAs, lncRNAs can also be detected in the sputum, blood, and urine of cancer patients, indicating that some lncRNAs may be potential non-invasive diagnosis targets for human cancers. For example, lncRNA PCA3, specifically expressed in the prostate, has been developed as an early diagnosis marker of prostate cancer, which is more sensitive and specific than serum prostate specific antigen (PSA) 10, 11. LncRNAs are also being prime targets for cancer therapy 5. LncRNAs appear to form a secondary structure and act as multicomponent complexes, making lncRNAs attractive for cancer therapeutic intervention 12. In addition, several other features, such as low abundance and tissue specificity, also support lncRNAs as potential efficacious anti-tumor targets. LncRNA H19, upregulated in many cancers, exerts oncogenic effects through promoting cancer progression, angiogenesis, and metastasis 13, 14. H19-DTA (BC-819) is a DNA plasmid that carries the diphtheria toxin gene under the regulation of the H19 promoter sequence and therefore has a potential therapeutic effect on a variety of tumors overexpressing H19 gene. Currently, BC-819 has been advanced into a series of phase I/IIb clinical trials for patients suffering from bladder, ovarian, and pancreatic cancer (https://www.clinicaltrials.gov/), and four completed clinical trials have demonstrated BC-819 is safe and feasible for tumor treatment 15-17. In short, lncRNAs are emerging as promising biomarkers and therapeutic targets in cancer.

Tumor progression is significantly attributable to a distinctive and suitable tumor microenvironment (TME), that is largely maintained by a variety of stromal cells including cancer stem cells (CSCs), cancer endothelial cells (CECs), cancer-associated fibroblasts (CAFs) and infiltrated immune cells 18, 19. Stromal cells are recruited and activated, acting together to trigger downstream signals that promote tumor formation, angiogenesis and metastasis 20, 21. This has attracted increasing attention aimed at identifying these stromal cells as potential targets for novel cancer therapies. Recently, numerous studies revealed that various lncRNAs play significant roles in the regulation of TME, particularly stromal cells. The main objective of this review is to discuss the basic properties and functional roles of the lncRNAs in the contribution of the TME, to lay a foundation for lncRNAs-based therapies in cancer treatment.

## LncRNAs play crucial roles in the modulation of the TME

### LncRNAs as modulators of infiltrated immune cells

**Figure 1:**
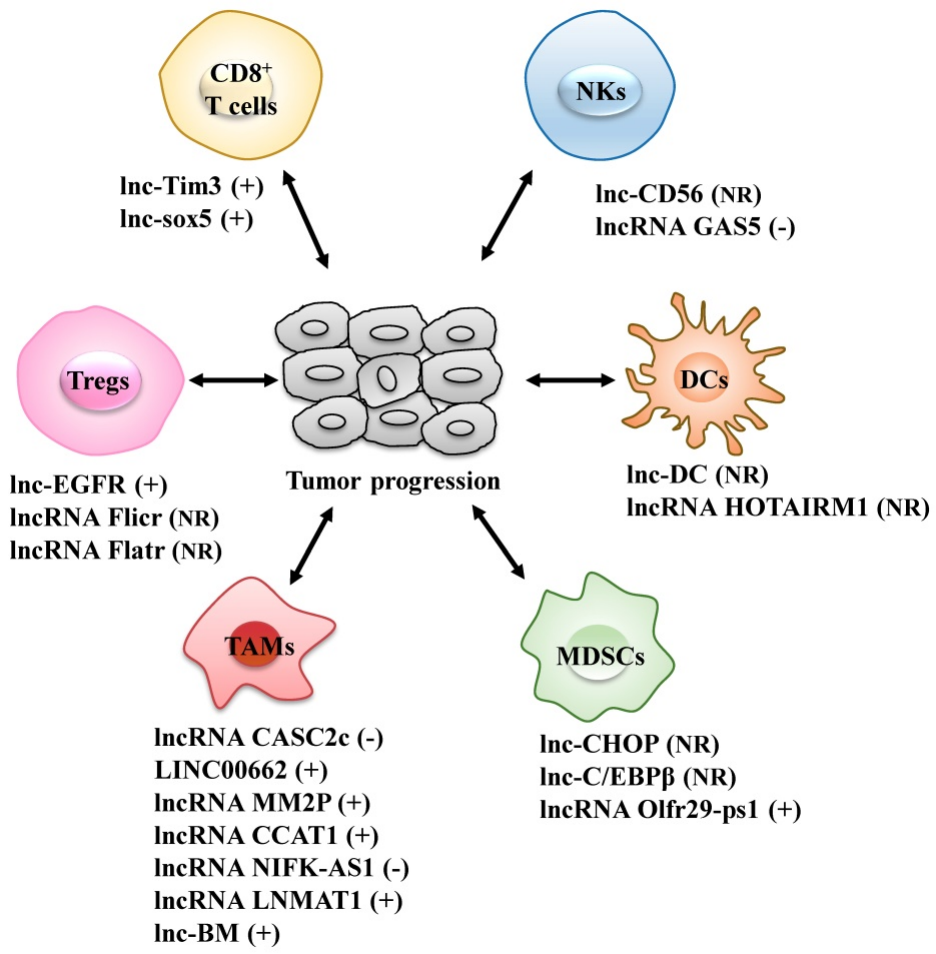
LncRNAs act as modulators between immune cells and tumor cells. Immune cells include CD8^+^ T cells, regulatory T cells (Tregs), dendritic cells (DCs), natural killer cells (NKs), tumor-associated macrophages (TAMs) and myeloid-derived suppressor cells (MDSCs). +: promoting the tumor progression; -: inhibiting the tumor progression; NR: Not Reported.

Different types of infiltrated immune cells are important components of the TME and act together to help cancer cells to escape immune surveillance, thus generating a tumor-promoting microenvironment for proliferation and metastasis of cancer cells 22. The roles of lncRNAs in the differentiation and function of various immune cells, including T cells, dendritic cells (DCs), natural killer cells (NKs), tumor-associated macrophages (TAMs) and myeloid-derived suppressor cells (MDSCs), are increasingly well understood (Fig. 1 and Table 1).

### LncRNAs as modulators of T cells

T cells, a predominant immune cell type in the TME, exert dual roles in tumor progression 23. Cancer cells exploit the immunosuppressive properties of T cells, while weakening the effective functions of anti-tumor T cells 24. LncRNAs have been recognized as important regulators of several T cell functions. Regulatory T cells (Tregs), an immunosuppressive subset of CD4^+^ T cells characterized by the expression of the master transcription factor forkhead box protein P3 (FOXP3), frequently accumulate in the TME and even represent the major population of infiltrating CD4^+^ T cells 25, 26. There is growing evidence that some lncRNAs, such as lnc-EGFR, Flicr and Flatr, are involved in Treg biology. Among them, lnc-EGFR is highly expressed in Tregs of patients with hepatocellular carcinoma (HCC), where it acts as an activator of Tregs differentiation. Mechanistically, lnc-EGFR binds specifically to EGFR, inhibits its ubiquitination and subsequent degradation, and sustains the activation of its downstream AP-1 and NF-AT1, two transcription factors for FOXP3, therefore leading to the enhancement of Tregs immunosuppressive function and promotion of HCC progression 27. Both Flicr and Flatr, two lncRNAs conserved and enriched in activated Tregs, were reported to play crucial roles in the regulation of FOXP3 expression and immunosuppressive function of Tregs 28, 29, but their roles in the TME remain unclear. These findings indicate that targeting specific lncRNAs in Tregs has a broad application prospect in the development of anti-tumor therapeutic strategies.

CD8^+^ T cells, major population of T cells in the TME, exert an efficient anti-tumor attack 30. LncRNAs such as lnc-Tim3 and lnc-sox5 participate in modulating the function of CD8^+^ T cells. The expression of lnc-Tim3 is up-regulated in HCC patients, which is negatively correlated with the production of IFN-γ and IL-2 by tumor-infiltrating CD8^+^ T cells. Mechanistically, lnc-Tim3 interacts with Tim-3 to release Bat3, and thereby suppresses downstream Lck/NF-AT1/AP-1 signaling, resulting in the exhaustion of CD8^+^ T cells and HCC immune evasion 31. Similarly, lnc-sox5 is significantly increased in colorectal cancer (CRC) and correlated with CRC progression. Extensively, lnc-sox5 knock-down dramatically promotes the infiltration and the cytotoxicity of CD8^+^ T by suppressing the expression of indoleamine 2,3-dioxygenase 1 (IDO1) and therefore suppresses the tumorigenesis of CRC 8. Collectively, these data suggest that dysregulations of lncRNAs in T cells affect immune evasion and tumor progression, and might be potential targets of tumor immunotherapy.

### LncRNAs as modulators of dendritic cells

Dendritic cells (DCs) in the TME exhibit an important role in cross-priming CD8^+^ T cells, in response to initiate and sustain anti-tumor T cells immunity 32-34. Notably, lncRNAs, such as lnc-DC and HOTAIRM1, have been implicated in the DCs differentiation. Wang *et al*. 35 showed that lnc-DC is exclusively expressed in conventional dendritic cells and driven by the transcription factor PU.1, a key regulator of DCs differentiation. Knockdown of lnc-DC down-regulates the expression of function-related genes and antigens uptake by DCs, thus impairing DCs differentiation from human monocytes and reducing their capacity to stimulate T cell activation. Mechanistic evidence has shown that lnc-DC exerts these effects by activating STAT3, a transcription factor that regulates DCs differentiation. Lnc-DC binds directly to the C terminus of STAT3 to block SHP1-mediated dephosphorylation of STAT3, thus promoting the phosphorylation of STAT3 on tyrosine-705 and the expression of genes associated with DCs activation. Subsequently, Xin *et al*. 36 reported that lncRNA HOTAIRM1 is downregulated during the process of monocyte differentiating into DCs. HOTAIRM1 competitively binds to miR-3960, hinders miR-3960 from repressing monocyte-related HOXA1 mRNA expression and induces subsequent upregulation of two monocyte markers CD14 and B7H2, thereby sustaining monocyte phenotype and blocking cells entry into the DCs differentiation pathway. However, the role during tumor progression of these lncRNAs within DCs awaits further investigation.

### LncRNAs as modulators of natural killer cells

Natural killer cells (NKs) play a critical role in the anti-tumor immune response and participate in controlling tumor progression and metastasis, due to their cytotoxic potential and ability to release immunoregulatory cytokines 37, 38. The typically studied lncRNA in the NKs is lnc-CD56, which is involved in the regulation of CD56, a classical human NKs surface marker. Knockdown of lnc-CD56 reduces the expression of CD56, suggesting lnc-CD56 may be a positive regulator of CD56 and essential for the development and diverse functions of NKs 39. Recently, Fang *et al*. 40 found that lncRNA GAS5 is down-regulated in NKs of patients with liver cancer, while up-regulated in activated NKs compared with non-stimulated NKs. Overexpression of GAS5 in activated NKs increases IFN-γ secretion, NKs cytotoxicity, and the percentage of CD107a^+^ NKs through regulating miR-544/RUNX3, hence, enhancing the killing effects of NKs and inhibiting tumor growth. Moreover, down-regulation of GAS5 is associated with liver cancer progression, conferring a worse overall patient survival 41. These findings highlight the importance of lncRNAs in NKs functions and anti-tumor immune response.

### LncRNAs as modulators of tumor-associated macrophages

**Figure 2:**
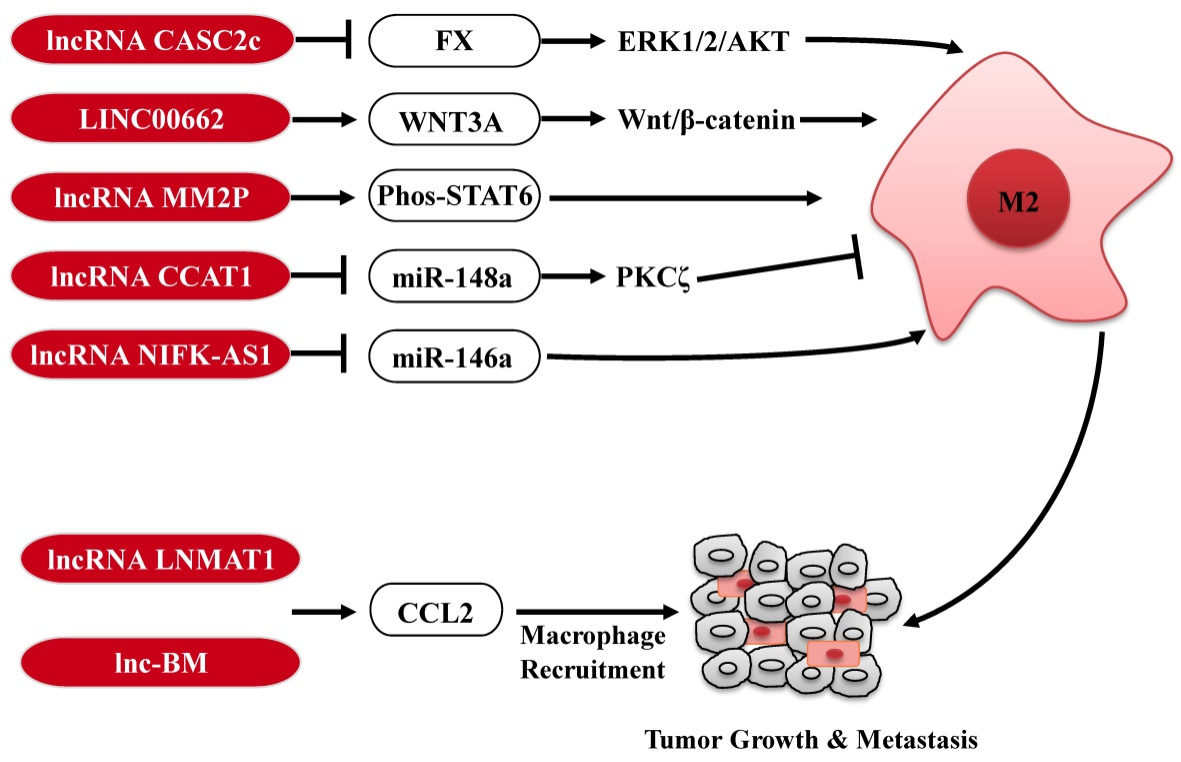
The regulatory roles of lncRNAs in TAMs. LncRNAs are involved in regulation the polarization of TAMs or CCL2-mediated macrophages recruitment to affect tumor progression. M2: M2 macrophage.

Tumor-associated macrophages (TAMs) are key regulators of the TME, and orchestrate various aspects of tumor progression. In response to microenvironmental signals, TAMs undergo the polarization of pro-inflammatory M1 or anti-inflammatory M2 and, therefore, have anti-tumor or pro-tumor abilities 42. Several lncRNAs expressed in tumor cells are involved in the polarization of TAMs to affect tumor progression (Fig. 2). For example, in glioblastoma multiforme, lncRNA CASC2c inhibits macrophage migration and polarization to the M2 subtype, via binding to coagulation factor X (FX) and commonly inhibiting its expression and secretion. The reduction of FX secreted in the tumor microenvironment results in the decrease of the phosphorylation and activation of ERK1/2 and AKT in macrophages, which plays a crucial role in the M2 macrophage polarization 43. In contrast, in HCC, lncRNA LINC00662 induces WNT3A expression and secretion, and consequently activates Wnt/β-catenin signaling in macrophages in a paracrine manner. Therefore, LINC00662 promotes M2 macrophage polarization and consequently results in HCC tumor growth and metastasis. Clinical data further confirm that high expression of LINC00662 in HCC is correlated with overactivated WNT3A, M2 macrophage polarization and poor prognosis of HCC patients 44. In addition, a high-throughput profiling shows that lncRNA-MM2P is specifically upregulated during the polarization of M2 macrophages. Knockdown of lncRNA-MM2P in macrophages reduces phosphorylation on STAT6, suppresses the transcription of M2-related genes in macrophages, and ultimately blocks M2 macrophage polarization. Thus, targeting lncRNA-MM2P impairs macrophage-promoted tumor angiogenesis and progression 45. Besides, lncRNAs, CCAT1 and NIFK-AS1 expressed in macrophages, also play a key role in modulating the polarization of TAMs in prostate cancer and endometrial cancer, respectively 46, 47.

Furthermore, tumor-derived CCL2 is released into the TME and recruits macrophages to promote tumor progression 48, 49. LncRNAs are also involved in regulation of TAMs infiltration in tumor through affecting CCL2 expression (Fig. 2). For example, lncRNA LNMAT1 is markedly upregulated in lymph node (LN)-metastatic bladder cancer and associated with LN metastasis and prognosis. Specifically, LNMAT1 epigenetically activates CCL2 transcription by enhancing hnRNPL-mediated histone H3 lysine 4 trimethylation (H3K4me3) at the CCL2 promoter. LNMAT1-induced CCL2 in bladder cancer cells contributes to recruit macrophages into the tumor and ultimately promotes lymphatic metastasis of bladder cancer 50. Similarly, high expression of lnc-BM also promotes breast cancer brain metastasis, through inducing STAT3-dependent expression of CCL2 to attract macrophages to the tumor 51. Taken together, lncRNAs expressed in TAMs or secreted by tumor cells modulate the function of TAMs through diverse mechanisms, further affecting tumorigenesis and metastasis, reminding us that targeting these lncRNAs in the TAMs or tumor cells may be a potential anti-tumor strategy.

### LncRNAs as modulators of myeloid-derived suppressor cells

Myeloid-derived suppressor cells (MDSCs) are the central cell population with potent immunosuppressive activity on T cells 52, 53. It has been demonstrated that some lncRNAs such as lnc-CHOP and lnc-C/EBPβ, are involved in the modulation of generation, recruitment and immunosuppressive functions of MDSCs. Lnc-CHOP interacts with CHOP and C/EBPβ isoform liver-enriched inhibitory protein (LIP) to promote the activation of C/EBPβ, and results in the expression of major molecules linked to MDSC immunosuppressive activity, including ARG1 and NOS2 54. In contrast, lnc-C/EBPβ was reported to bind with LIP to inhibit the activation of C/EBPβ, and further reduce immunosuppressive function and differentiation of MDSCs 55. A recent study showed that lncRNA Olfr29-ps1, is expressed in MDSCs and upregulated by the proinflammatory cytokine IL6. Olfr29-ps1 promotes immunosuppressive function and differentiation of monocytic MDSCs, through a N^6^-methyladenosine (m6A)-modified regulatory network 56. These findings reveal diverse mechanisms by which lncRNAs regulate the function of MDSCs and also provide potential therapeutic targets.

### LncRNA as modulators of cancer stem cells

**Figure 3:**
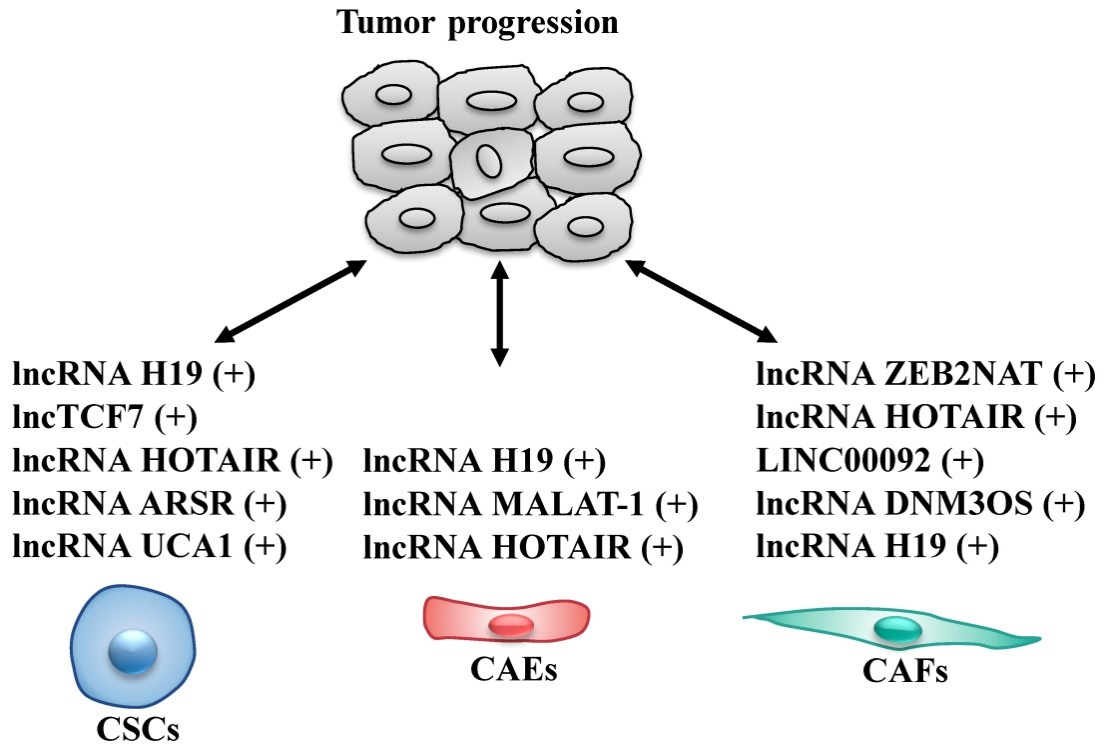
LncRNAs act as modulators between cancer stem cells (CSCs)/cancer-associated endothelial cells (CAEs)/cancer-associated fibroblasts (CAFs) and tumor cells. +: promoting the tumor progression.

Cancer stem cells (CSCs), a rare sub-population within tumor, are key components of the TME, that have the ability of self-renewal and limitless proliferation 57. CSCs are believed to be responsible for tumor initiation, progression and resistance to therapies 58, 59. Several studies described that various lncRNAs are able to modulate self-renewal, maintenance and differentiation of CSCs through different molecular mechanisms (Fig. 3 and Table 2). To date, lncH19, lncTCF7, lncARSR, UCA1 and HOTAIR are the most highlighted lncRNAs in CSCs.

lncRNA H19 is one of the first non-coding RNAs identified as a cancer-related lncRNA 60. In prostate cancer, H19 level modulations are positively correlated with the expression of key pluripotency transcription factors (*Oct4*, *Sox2*) and the sphere-forming capacity, uncovering a role for H19 as a potential stemness regulator 61. In breast cancer, H19 acts to sponge miRNA let-7 and inhibit its biological function, subsequently leading to the elevation of LIN28, the core pluripotency factor that is crucial for the maintenance of breast cancer stem cells (BCSCs) 62. In HCC, lncTCF7 recruits the SWI/SNF chromatin remodeling complex to TCF7 promoter to regulate TCF7 transcription, activating the Wnt signaling cascade and thus priming liver CSCs self-renewal and tumor propagation 63.

Hox transcript antisense intergenic RNA (HOTAIR) is an oncogenic lncRNA, the expression of which is elevated in multiple CSCs 64-66, and positively associated with advanced tumor progression and poor prognosis 67. HOTAIR is able to promote liver cancer stem cell malignant growth through downregulation of SETD2, a specific methyltransferase for histone H3 lysine 36 (H3K36me3) and required for ATM activation upon DNA double-strand breaks (DSBs) 68. Additionally, HOTAIR is highly up-regulated in BCSCs models, and tightly regulates self-renewal capacity of CSCs through transcriptional inhibition of miR-34a and consequent upregulation of Sox2 65.

The lncRNA activated in renal cell carcinoma with sunitinib resistance (ARSR) was recently identified as a novel lncRNA. ARSR is highly expressed in primary renal CSCs and predicts poor prognosis. ARSR processes self-renewal capacity and promotes the metastasis of renal CSCs. Mechanistically, ARSR interacts with Yes-associated protein (YAP) to block its phosphorylation by LATS1, thus facilitating YAP nuclear translocation, which is required to sustain CSCs self-renewal 69-71.

The lncRNA urothelial cancer associated 1 (UCA1) is highly expressed in multiple human cancers, including hepatocellular cancer, gastric cancer, colorectal cancer and lung cancer, which confers a worse overall patient survival 72-77. Notably, UCA1 plays a critical role in governing growth and malignant transformation of CSCs through the upregulation and activation of telomerase reverse transcriptase (TERT) and oncogene C-myc. Mechanistically, excessive UCA1 leads to increased binding capacity of UCA1 to CyclinD1. Therefore, UCA1-CyclinD1 complex is recruited to c-Myc promoter region, increasing the outcome of oncogene C-myc 78. On the other hand, UCA1-CyclinD1 complex also activates lncRNA H19 transcription via reducing DNA methylation on H19 promoter region. Strikingly, overexpression of H19 enhances the binding of TERT, thus enhancing the cell telomerase activity and extending the telomere length 78. Besides, UCA1 enhances the phosphorylation of RB1, which could promote the interplay between histone lysine methyltransferase SET1A and pRB1. Then, the complex induces the trimethylation of H3K4 on telomere capping essential gene TRF2 promoter region, causing TRF2 overexpression and consequently prolonging the telomere length 79. Taken together, these observations highlight the critical role of multiple lncRNAs in modulating CSCs maintenance and self-renewal and provide a potential application for targeting lncRNAs as an alternative effective strategy.

### LncRNAs as modulators of cancer-associated endothelial cells

Cancer-associated endothelial cells (CAEs), important components of the TME, are responsible for angiogenesis and regulation of tumor growth and metastasis 80, 81. Altered expression of lncRNAs in CAEs also affects tumor progression through modulating the biological behaviors of CAEs (Fig. 3 and Table 2).

H19 expression is significantly up-regulated in glioma microvessels and glioma-induced endothelial cells (GECs). H19 regulates the proliferation, migration and tube formation of GECs by targeting miR-29a, which decreases the expression of vasohibin 2 (VASH2), an angiogenic factor 82. H19 derived from tumor cells could also affect angiogenesis of CAEs. CD90^+^ liver cancer cells package H19 inside exosomes, which is released to influence endothelial cells by promoting angiogenesis and stimulating their adhesive properties 83.

MALAT1 can also regulate the properties of CAEs. Researchers have found that knockdown of MALAT1 expression significantly inhibits the proliferation of human umbilical vein endothelial cells (HUVECs), which is mediated by upregulation of miR-320a and hence, downregulation of cell cycle regulator FOMX1 expression in HUVECs 84. In neuroblastoma, up-regulation of MALAT1, induced by hypoxia, results in endothelial cell migration, invasion and vasculature formation via increasing the expression of the pro-angiogenic factor, FGF2 85, 86. These data demonstrate that MALAT1 plays an important role in endothelial cell proliferation and tumor angiogenesis.

HOTAIR is extremely abundant in nasopharyngeal carcinoma cells (NPC) and functions as an angiogenesis activator. Specifically, HOTAIR promotes endothelial cell tube formation and angiogenesis through directly activating the transcription of angiogenic factor VEGFA 87. Furthermore, in glioma cells, the angiogenic function of HOTAIR is mediated not only by the regulation of VEGFA expression, but also by direct transmission into endothelial cells via glioma cell-derived vesicles 88. Collectively, deregulated lncRNAs expressions in CAEs do affect the tumor progression, suggesting that targeting lncRNAs both in CAEs and tumor cells might be a new approach for cancer therapy.

### LncRNA as modulators of cancer-associated fibroblasts

Cancer-associated fibroblasts (CAFs), the activated fibroblasts, are one of the most dominant components of the tumor microenvironment 89, 90. CAFs are able to promote tumor progression, such as proliferation, invasion and angiogenesis, through the secretion of growth factors, cytokines and chemokines 91. Although the role of lncRNA in CAFs modulation is poorly investigated, some studies suggested that they can contribute to: i) CAFs phenotype and function; ii) enhancement of CAFs-triggering signals (e.g. TGF-β1) to induce epithelial-mesenchymal transition (EMT) and metastasis of tumor cells (Fig. 3 and Table 2). High-throughput sequencing technologies reveal numerous novel lncRNAs differentially expressed in distinguishing CAFs from normal ovarian fibroblasts (NOFs), followed by the functional network to predict those specific lncRNAs involved in the pro-metastatic phenotype of CAFs 92.

TGF-β1 secreted by CAFs induces the metastatic activity of cancer cells by regulating the expression of lncRNAs. For example, CAFs-mediated upregulation of lncRNA ZEB2NAT transcription in bladder cancer cells that promotes EMT via the secretion of TGF-β1, which is linked to poor clinical outcome 93. Similarly, CAFs-secreted TGF-β1 induces the transcription of lncRNA HOTAIR to promote EMT and metastasis in breast cancer cells 94. Moreover, the lncRNA, LINC00092, is induced upon stimulation by CAF-secreted CXCL14 in ovarian cancer and correlated with poor prognosis in patients. Mechanistically, LINC00092 binds with fructose-2, 6-biphosphatase 2 (PFKFB2), thereby promoting ovarian cancer metastasis by altering glycolysis and sustaining CAFs-like features of fibroblasts 95.

In addition, CAF-promoted lncRNAs are also involved in radio-resistance and chemo-resistance. In esophageal cancer cells, CAFs-promoted expression of lncRNA DNM3OS confers significant radio-resistance via regulating DNA damage response in a FOXO1-dependent manner 96. Moreover, lncRNA H19 expressed by CAFs was reported to contribute to chemo-resistance of colorectal cancer through activating the β-catenin pathway 97. Collectively, these studies indicate the importance of lncRNAs in the interaction between the CAFs and cancer cells, reminding us that targeting lncRNAs could be a new approach for cancer therapy.

## Conclusion and further perspective

Here, we have summarized recent advancement involving of lncRNAs within the tumor microenvironment and their roles in the crosstalk between infiltrated immune cells, CSCs, CAEs, CAFs and tumor cells, as well as some of the underlying molecular mechanisms. lncRNAs exert their functions in different ways to modulate tumor growth and progression. As yet, only a few lncRNAs have been well-studied in the tumor-stroma crosstalk, warranting further studies on the identification of more new types of lncRNAs and their mechanisms involved in the future. A better understanding of the role of lncRNAs within the tumor microenvironment may lead to the discovery of potential biomarkers and development of novel targeted therapies.

At present, the pressing issue is to systematically elucidate the key aspects of lncRNAs, including the expression, structure, function and regulatory mechanism. The improvement of analytical technologies for the specific biological functions of lncRNAs may help explore its greatest relevance to various cancers. Such information will provide a basis for considering lncRNAs as ideal diagnostic markers or even therapeutic targets. In addition, approaches of targeting lncRNAs should be considered and optimized, such as the use of siRNA to induce lncRNA degradation and CRISPR/Cas9 mediated gene editing. How to specifically deliver the respective molecules into targeted cells is still a great challenge.

In summary, the functional importance of lncRNAs within tumor microenvironment is gradually characterized; clinical application of lncRNAs still needs to be studied further. With the deep-going research, lncRNAs-associated tumor-stroma crosstalk will open up a new era of anti-tumor therapy.

## Figures and Tables

**Table 1:** LncRNAs act as modulators between immune cells and tumor cells

LncRNA	Cancer type	Stromal cells	Mechanism of action	Ref.
lnc-EGFR	Hepatocellular carcinoma	Tregs	Lnc-EGFR binds to EGFR, and activates AP-1/NF-AT1/FOXP3 axis, promoting Tregs differentiation and tumor progression	27
Flicr	-	Tregs	Flicr inhibits the expression of FOXP3 and curtails the immunosuppressive function of Tregs	28
Flatr	-	Tregs	Flatr promotes the expression of FOXP3 and enhances the immunosuppressive function of Tregs	29
lnc-Tim3	Hepatocellular carcinoma	CD8^+^ T	Lnc-Tim interacts with Tim-3 to release Bat3 and induces exhaustion of CD8^+^ T cells, exerting HCC immune evasion	31
lnc-sox5	Colorectal cancer	CD8^+^ T	Lnc-sox5 suppresses the infiltration and the cytotoxicity of CD8^+^ T by increasing IDO1 expression and therefore promotes the tumorigenesis	8
lnc-DC	-	DCs	Lnc-DC binds directly to the C terminus of STAT3 and activates it, thus promoting DCs differentiation.	35
HOTAIRM1	-	DCs	HOTAIRM1 induces upregulation of CD14 and B7H2, thus blocking cells to enter into the DC differentiation pathway	36
lnc-CD56	-	NKs	Lnc-CD56 may function as a positive regulator of CD56, and be essential for the developmental and diverse functions of NKs	39
GAS5	Liver cancer	NKs	GAS5 regulates miR-544/RUNX3 to enhance the killing effects of NKs, leading to inhibition of tumor growth.	40
CASC2c	Glioblastoma multiforme	TAMs	CASC2c suppresses the M2 polarization by repressing expression of FX in GBM cells and inhibiting ERK1/2 and AKT in macrophages.	43
LINC00662	Hepatocellular carcinoma	TAMs	LINC00662 activates Wnt/β-catenin signaling and further promotes M2 polarization, promoting tumor growth and metastasis.	44
MM2P	Osteosarcoma	TAMs	MM2P promotes M2 polarization by inducing phosphorylation on STAT6, resulting in macrophage-promoted tumor progression.	45
CCAT1	Prostate cancer	TAMs	CCAT1/miR-148a/PKCζ prevents cell migration of prostate cancer by altering macrophage polarization.	46
NIFK-AS1	Endometrial cancer	TAMs	NIFK-AS1 inhibits M2 polarization of macrophages and malignant phenotype of endometrial cancer cells through targeting miR-146a.	47
LNMAT1	Bladder cancer	TAMs	LNMAT1 activates CCL2 expression to recruit macrophages into the tumor and ultimately promote lymphatic metastasis of bladder cancer.	50
lnc-BM	Breast cancer	TAMs	lnc-BM induces STAT3-dependent expression of CCL2 to attract macrophages, promoting breast cancer brain metastasis.	51
lnc-CHOP	-	MDSCs	Lnc-CHOP upregulates the expression of ARG1 and NOS2 to enhance the immunosuppressive function of MDSCs.	54
lnc-C/EBPβ	-	MDSCs	lnc-C/EBPβ inhibits the expressions of ARG1 and NOS2, to suppress immune-suppressive function and differentiation of MDSCs.	55
Olfr29-ps1	Melanoma	MDSCs	Olfr29-ps1promotes functions of monocytic MDSCs and thus tumor growth, through a m6A-modified regulatory network.	56

Tregs: regulatory T cells; DCs: dendritic cells; NKs: natural killer cells; TAMs: tumor-associated macrophages; MDSCs: myeloid-derived suppressor cells.

**Table 2:** LncRNAs act as modulators between cancer stem cells (CSCs)/cancer-associated endothelial cells (CAEs)/cancer-associated fibroblasts (CAFs) and tumor cells

LncRNA	Cancer type	Stromal cells	Mechanism of action	Ref.
H19	Prostate cancer	CSCs	H19 increases the expression of Oct4 and Sox2 to promote the sphere-forming capacity.	61
H19	Breast cancer	CSCs	H19 sponges and inhibits miRNA let-7 and enhances the expression of LIN28, contributing to the maintenance of stem cells.	62
lncTCF7	Hepatocellular carcinoma	CSCs	lncTCF7 activates the Wnt signaling cascade to prime CSC self-renewal and tumor propagation.	63
HOTAIR	Liver cancer	CSCs	HOTAIR enhances liver CSC growth through dissociating the CREB-p300-RNApolII complex to repress expression of SETD2.	68
HOTAIR	Breast cancer	CSCs	HOTAIR maintains BCSCs self-renewal capacity by negatively regulating miR-34a and consequently Sox2.	65
ARSR	Renal carcinoma	CSCs	ARSR promotes the expansion of renal CSCs through interaction with YAP, to block its phosphorylation.	69-71
UCA1	Liver cancer	CSCs	UCA1 promotes the malignant transformation of hepatocyte-like stem cells via activating telomere length extension and c-Myc expression.	78
H19	Glioma	CAEs	H19 promotes glioma-induced endothelial cell proliferation, migration and tube formation via increasing the expression of VASH2.	82
H19	Liver cancer	CAEs	CD90^+^ liver cancer cells package H19 inside exosomes, which is released to promote functions of endothelial cells.	83
MALAT-1	-	CAEs	MALAT-1 promotes the proliferation rate of HUVECs by upregulation of FOMX1 expression.	84
MALAT-1	Neuroblastoma	CAEs	MALAT1 promotes endothelial cell migration, invasion and vasculature formation via increasing the expression of FGF2.	85,86
HOTAIR	Nasopharyngeal carcinoma	CAEs	HOTAIR promotes endothelial cell tube formation and angiogenesis through activating the transcription of VEGFA and Ang2 expression.	87
HOTAIR	Glioma	CAEs	HOTAIR promotes the angiogenic function by activating VEGFA and glioma cell-derived vesicles.	88
ZEB2NAT	Bladder cancer	CAFs	ZEB2NAT promotes the CAFs-mediated initiation of efficient metastasis in a TGFβ1-dependent process.	93
HOTAIR	Breast cancer	CAFs	HOTAIR promotes the CAFs-mediated tumor development in a TGFβ1-dependent process.	94
LINC00092	Ovarian cancer	CAFs	CAFs-secrete CXCL14 induces LINC00092 upregulation to promote tumor metastasis by enhancing PFKFB-2 translation.	95
DNM3OS	Esophageal cancer	CAFs	CAFs-promoted expression of DNM3OS confers significant radio-resistance via regulating DNA damage response.	96
H19	Colorectal cancer	CAFs	H19 expressed by CAFs contributes to chemo-resistance of colorectal cancer through activating the β-catenin pathway.	97

## Abbreviations

AKT: AKT serine/threonine kinase 1
ARG1: arginase-1
ARSR: activated in renal cell carcinoma with sunitinib resistance
BCSC: breast cancer stem cell
C/EBPβ: CCAAT enhancer binding protein beta
CAE: cancer-associated endothelial cell
CAF: cancer-associated fibroblast
CASC2c: CASC2 cancer susceptibility 2
CCAT-1: colon cancer‐associated transcript‐1
CCL2: C-C motif chemokine ligand 2
CHOP: C/EBP homologous protein
CRC: colorectal cancer
CSC: cancer stem cell
CXCL14: C-X-C motif chemokine ligand 14
DC: dendritic ell
DSB: DNA double-strand break
EGFR: epidermal growth factor receptor
EMT: epithelial-mesenchymal transition
ERK1/2: extracellular signal-related kinase 1/2
FGF2: fibroblast growth factor 2
Flatr: Foxp3-specific lncRNA anticipatory of Tregs
Flicr: FOXP3 regulating long intergenic non-coding RNA
FOMX1: forkhead box M1
FOXO1: forkhead box O1
FOXP3: forkhead box protein P3
GAS5: growth arrest specific 5
GEC: glioma-induced endothelial cells
HCC: hepatocellular carcinoma
hnRNPL: heterogeneous nuclear ribonucleoprotein L
HOTAIR: HOX transcript antisense RNA
HOTAIRM1: HOXA transcript antisense RNA, myeloid-specific 1
HOXA1: homeobox A1
HUVEC: human umbilical vein endothelial cell
IDO1: indoleamine 2,3-dioxygenase 1
IFN-γ: interferon gamma
IL-2: interleukin 2
IL-6: interleukin 6
LATST1: large tumor suppressor kinase 1
LIN28: lin-28 homolog A
LIP: liver-enriched inhibitory protein
lncRNAs: long noncoding RNAs
LNMAT1: lymph node metastasis associated transcript 1
MALAT1: metastasis associated lung adenocarcinoma transcript 1
MDSC: myeloid-derived suppressor cell
NIFK-AS1: NIFK antisense RNA 1
NK: natural killer
NOS2: NO synthase 2
NPC: nasopharyngeal carcinoma cell
PCA3: prostate cancer associated 3
PFKFB2: fructose-2, 6-biphosphatase 2
RB1: RB transcriptional corepressor 1
RCC: renal cell carcinoma
RUNX3: RUNX family transcription factor 3
SET1A: SET domain containing 1A, histone lysine methyltransferase
SETD2: SET domain containing 2, histone lysine methyltransferase
STAT3: signal transducer and activator of transcription 3
STAT6: signal transducer and activator of transcription 6
TAM: tumor-associated macrophage
TERT: telomerase reverse transcriptase
TGF-β: transforming growth factor beta
TME: tumor microenvironment
Treg: regulatory T cell
TRF2: TATA box binding protein-related factor 2
UCA1: urothelial cancer associated 1
VASH2: vasohibin 2
VEGFA: vascular endothelial growth factor A
WNT3A: Wnt family member 3A
YAP: Yes-associated protein
